# Long-term outcome of bail-out ViV-TAVI at index procedure

**DOI:** 10.1007/s00392-025-02640-5

**Published:** 2025-04-29

**Authors:** Isabel Horn, Hazem Omran, Sabine Bleiziffer, Smita Scholtz, Kai Friedrichs, Cornelia Piper, Johanna Bormann, Sara Waezsada, Max Potratz, René Schramm, Volker Rudolph, Tanja K. Rudolph

**Affiliations:** 1 Klinik für Allgemeine und Interventionelle Kardiologie/Angiologie, Herz- und Diabeteszentrum NRW, Georgstr. 11, 32545 Bad Oeynhausen, Germany; 2 Clinic for Thoracic and Cardiovascular Surgery, Herz- Und Diabeteszentrum NRW, Ruhr-Universität Bochum, Bad Oeynhausen, Germany

**Keywords:** Aortic stenosis, Bailout, Migration, Embolization, Valve-in-valve, TAVI, Conversion

## Abstract

**Objective:**

This study aimed to compare in-hospital and long-term outcomes of patients with bail-out valve-in-valve TAVI due to a primarily failed transcatheter aortic valves procedure (bViV-TAVI) versus a successful transcatheter aortic valve implantation (TAVI).

**Methods:**

We recorded bViV-TAVI procedures at our center from February 2011 to March 2022. Primary endpoint was long-term mortality. In-hospital mortality, stroke, acute kidney failure, need for new permanent pacemaker, and duration of intervention were secondary endpoints.

**Results:**

4555 patients undergoing TAVI were retrospectively included. 231 matched (77:154) patients were analyzed. BViV-TAVI was a rare event (1.9%). In 76.7% of the cases transcatheter valve embolization and migration were the reasons for implanting a second valve in the same procedure. Significant PVL accounted for bViV-TAVI in 23.4% of the patients. The duration of the intervention was significantly longer for the bViV-TAVI group (*p* < 0.001). BViV-TAVI patients showed higher rates of a new permanent pacemaker implantation (*p* = 0.013) and the postprocedural mean pressure was significantly higher (*p* = 0.03). Concerning the other secondary endpoints there was a trend for a higher event rate in bVIV-TAVI patients which did not reach significant difference. After an average follow-up period of 4.9 ± 3.0 years, mortality was significantly higher in the bViV-TAVI group (54.5% vs. 39.0%, *p* = 0.025).

**Conclusion:**

The implantation of a second valve during the same procedure as bail-out is a feasible alternative treatment option in patients with failed transcatheter aortic valve procedures. However, increased long-term mortality must be taken into account.

**Graphical abstract:**

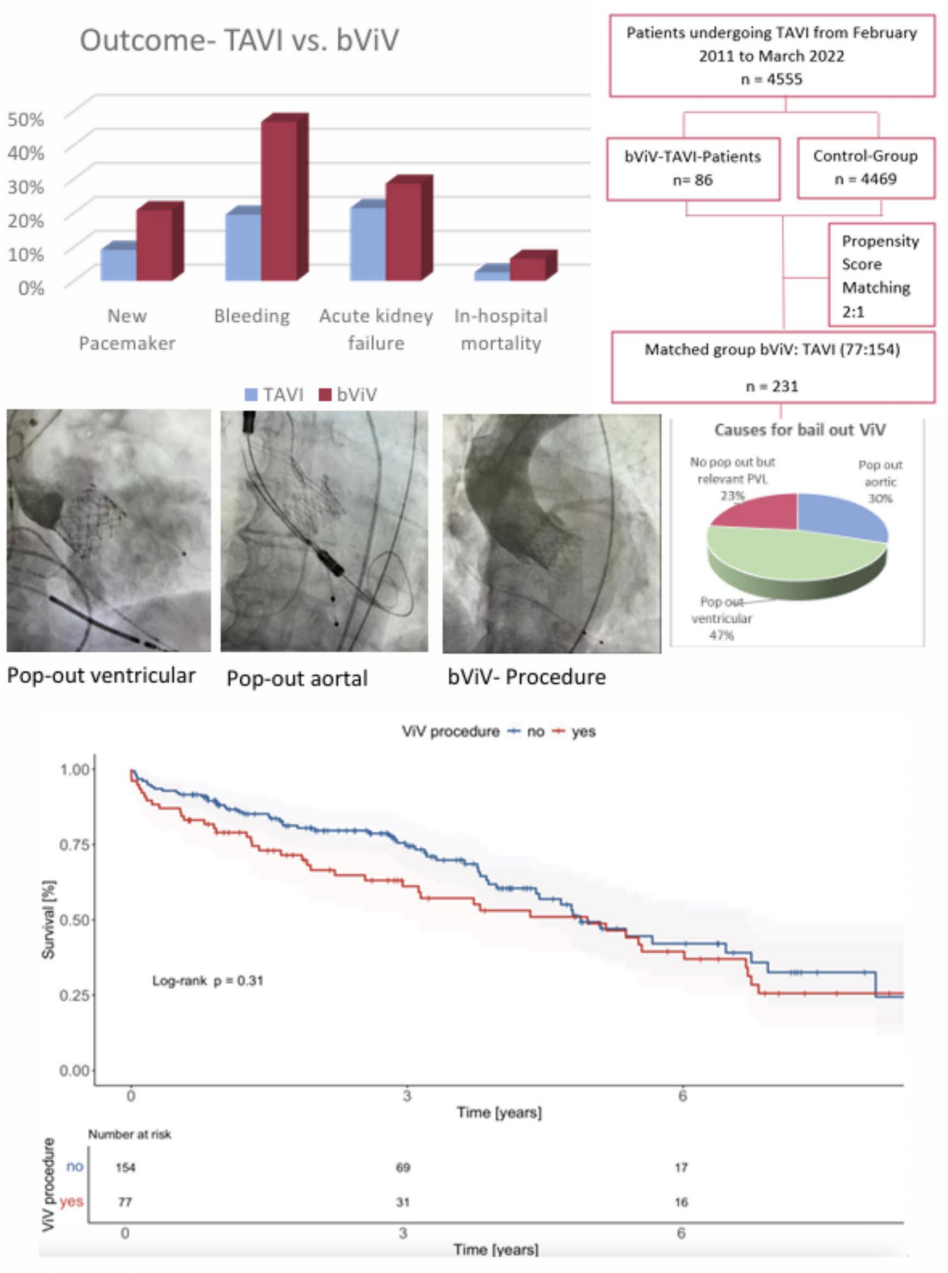

**Supplementary Information:**

The online version contains supplementary material available at 10.1007/s00392-025-02640-5.

## Introduction

Over the past years, transcatheter aortic valve implantation (TAVI) was set up as a standard treatment for older patients or patients with increased operative risk [1, 2].

A broad range of studies comparing outcomes of TAVI versus surgical aortic valve replacement (SAVR) has shown that TAVI in patients with high or intermediate operative risk is non-inferior [2–4]. Furthermore, even in patients with lower surgical risk, TAVI was non-inferior to surgery [5, 6] also taking into consideration long-term outcomes [7–10]. Although growing experience and improved pre-procedural planning reduced peri-procedural TAVI complications, bail-out strategies are always needed especially considering the fast development of TAVI implantations. As opposed to SAVR the anchoring of the TAVI device is remote and indirect which poses a higher risk for valve migration, embolization and paravalvular leakage (PVL).

Only a few case reports [11, 12] and studies have elaborated outcomes of bail-out strategies for failed TAVI-procedures [13–16]. Most of the reports are limited to a short follow-up period. The current study aimed to compare short- and long-term outcomes of patients with primarily failed transcatheter aortic valve procedures due to valve migration, embolization or a significant PVL treated with the implantation of a second valve (bViV-TAVI) as bail-out strategy versus primarily successful TAVI.

## Methods

### Study design and population

In this retrospective, single center registry, data for all patients undergoing TAVI from February 2011 to March 2022 were included. Follow-up time for the primary endpoint ended in September 2022. The inclusion criterion for the bViV-TAVI group was a bail-out implantation of a second valve during the same procedure. The indication for TAVI and the access was set by an interdisciplinary heart-team of cardiologists and cardiac surgeons. The type and size of the implanted valve were at the operator´s discretion, based on a computed tomography and echocardiographic imaging.

Propensity score matching was performed to adjust for baseline characteristics (age, gender, LV-EF, EuroSCORE II and COPD) between the bViV-TAVI and the TAVI group. The study protocol was approved by the local Ethics Committee of the Ruhr University Bochum (Germany) and carried out in accordance with the Declaration of Helsinki.

### Definitions

Primary endpoint was long-term all-cause mortality of all patients included from February 2011 to September 2022. In-hospital mortality, stroke, acute kidney failure, need for permanent new pacemaker, duration of the intervention, bleeding and vascular complications were secondary endpoints, which were defined according to the valve academic research consortium 3 (VARC- 3) criteria [17].

The causes of valve embolization and migration (TVEM) and significant PVL were documented by fluoroscopy or echocardiography and were recorded by the interventional report.

For follow-up, we collected data through outpatient visits, death registry queries and last available medical records.

### Statistical analysis

We performed a propensity score matching to adjust for baseline characteristics. The matching parameters included age, gender, LV-EF, EuroSCORE II and COPD.

Continuous variables were tested for normal distribution and evaluated as mean and standard deviation, categorical data were described as absolute numbers and percentage (%).

As continuous variables were not normally distributed, statistical analysis was based on unpaired samples and compared using the Mann–Whitney-*U*-Test. We used the Chi-quadrat-test or the Fisher’s exact test for categorical variables. Survival analysis was conducted using the Kaplan–Meier method and the log-rank test. A *p*-value < 0.05 was considered as statistically significant. For revealing independent risk factors, we used a hazard ratio. The calculations were conducted with IBM SPSS (Version 29.0.0.1, New York, USA).

## Results

4555 patients undergoing TAVI at our center were retrospectively included from February 2011 to March 2022, including 86 bail-out ViV TAVI-patients, which accounts for 1.9%.

Baseline characteristics of both unmatched cohorts are shown in the supplement (Table S1). 231 matched patients (1:2) were analyzed. After propensity score matching there was no difference in age (81.5 ± 6.1 vs. 82.44 ± 4.5), gender (48.1% vs. 53.9% female), body mass index (26.9 ± 4.6 vs. 26.9 ± 4.9). EuroSCORE II and Society of Thoracic Surgeons (STS) -Score were significantly higher in the bVIV-TAVI group (EuroSCORE II: 7.3 ± 8.8 vs. 6.0 ± 6.1, *p* = 0.023; STS-Score: 6.5 ± 6.1 vs. 5.2 ± 3.5, *p* = 0.012). Significantly more patients in the bViV-TAVI group had arterial hypertension (96.1% vs. 92.21%, *p* = 0.001). Baseline creatinine was significantly higher in the bVIV-TAVI group (1.4 ± 1.0 vs. 1.2 ± 0.7, *p* = 0.002). All other baseline characteristics were comparable in both groups (Table 1).

**Table 1:** Baseline characteristics

Features	Control	Control absolute	bViV	bViV absolute	*p*-value
Patients	154	154	77	77	
Age (years)	82.44 ± 4.5		81.5 ± 6.1		0.320
Female	53.9%	83	48.1%	37	0.402
Body mass index (kg/m^2^)	26.9 ± 4.9		26.9 ± 4.6		0.89
Hypertension	92.2%	124	96.1%	74	0.001
COPD	6.5%	10	14.3%	11	0.052
*New York Heart Association class*					
I	0,65%	1	1.3%	1	0.616
II	21.4%	33	27.3%	21	0.322
III	74.7%	115	66.2%	51	0.179
IV	3.3%	5	5.2%	4	0.471
Atrial fibrillation	38.3%	59	28.6%	22	0.144
Coronary artery disease	55.8%	86	67.5%	52	0.088
Previous cardiac surgery	16.9%	26	23.4%	18	0.236
Peripheral artery disease	9.1%	14	19.5%	15	0.025
Pre-existing cerebrovascular disease	7.8%	12	3.9%	3	0.257
Pre-existing pacemaker/ICD	13.0%	20	5.2%	4	0.067
STS Score (%)	5.2 ± 3.5		6.5 ± 6.1		0.012
EuroSCORE (%)	19.7 ± 15.6		22.7 ± 14.8		0.053
EuroSCORE II (%)	6.0 ± 6.1		7.3 ± 8.8		0.023
Baseline EF (%)	52.1 ± 8.3		51.5 ± 9.7		0.369
Baseline creatinine (mmol/l)	1.2 ± 0.7		1.4 ± 1.0		0.002
Pre CRP	1.1 ± 2.8		1.2 ± 2.8		0.301
Pre Hb	12.4 ± 1.7		12.7 ± 1.9		0.354
Annulus diameter	24.1 ± 2.5		26.6 ± 2.5		0.602

The self-expanding Core Valve Evolut TAVI valve was the most frequently implanted device in both groups (44.2% vs. 47.4%) followed by the balloon-expandable SAPIEN valve (33.8% vs. 26.6%) and the self-expanding ACURATE TAVI valve. The femoral access was the pre-dominant access in both groups (76.6% vs. 85.1%) followed by transapical access (20.8% vs. 13.6%) (Table 2).

**Table 2:** Procedural data

Features	Control	Absolut control	bViV-TAVI	Absolut bViV-TAVI	*p*-value
*Access*					
Aortic	1.3%	2	0%	0	0.315
Apical	13.6%	21	20.8%	16	0.115
Subclavian	0.0%	0	2.6%	2	0.110
Femoral	85.1%	131	76.6%	59	0.113
*First device*					
Acurate	20.1%	31	20.8%	16	0.908
CoreValve evolut	47.4%	73	44.2%	34	0.641
Sapien	26.6%	41	33.8%	26	0.259
Jena valve	1.3%	2	1.3%	1	1.000
Direct flow	2.6%	4	0%	0	0.154
Medtronic engager	1.9%	3	0%	0	0.294
First device balloon-expandable	26.6%	41	33.8%	26	0.259
First device size (mm)	26.9 ± 3.7		26.7 ± 2.6		0.617
Amount of contrast medium	104.8 ml ± 39.3		163.8 ml ± 66.1		< 0.001
Duration of the intervention (min)	71.3 ± 32.8		111.8 ± 46.0		< 0.001

In 76.7% of the cases, transcatheter valve embolization and migration were the reasons for implanting a second valve in the same procedure. Significant PVL accounted for bViV TAVI in 23.4% of the patients. Considering only the TVEM cases, ventricular pop-out occurred more often than aortic pop-out (61% vs. 39%). Embolization without an overlap between the primary and the secondary implanted valve occurred more often in aortic embolization than ventricular embolization (25.4% vs. 1.7%). Snaring was done in 20.8% of the cases. In 77.9%, the second valve was implanted with contact to the first valve. Complete ventricular embolization resulted in conversion to surgery in 100%.

For the second valve, a balloon-expandable TAVI device (SapienXT or 3) was the most frequently chosen valve (64.9%) followed by CoreValve/Evolut (29.9%) and Acurate (5.2%). In 74% of cases, the second device was from the same brand as the first device (Table 3).

**Table 3:** bViV-TAVI cohort

Features	bViV-TAVI	Absolut bViV-TAVI
Snaring done	20.8%	16
No overlap first and second device	22.1%	17
*Second device*		
Sapien	64.9%	50
Evolut/CoreValve	29.9%	23
Symetis	5.2%	4
Second device different from the first one	26.0%	20
Second device size (mm)	26.3 ± 2.4	
Pop out aortic	29.9%	23
Pop out ventricular	46.8%	36
No pop out but relevant PVL	23.3%	18
Aortic embolization	19.5%	15
Ventricular embolization	1.3%	1
Conversion	2.6%	2

The duration of the intervention was significantly longer for the bViV-TAVI group (111.8 min ± 46.0 vs. 71.3 ± 32.8, *p* < 0.001) and more contrast medium was used (163.8 ml ± 66.1 vs. 104.8 ml ± 39.3, *p* < 0.001).

There were two cases of conversion (2.6%) to open surgery in the bViV-TAVI group as opposed to none in the control group. Difficulties in positioning the TAVI prosthesis during the procedure via a transapical access led to cardiac arrest with necessity of an extracorporeal circulation and conversion to open surgery in one case. In the second case, the prosthesis embolized into the left ventricle and had to be removed by open surgery.

Regarding the clinical events of in-hospital death (6.5% vs 2.5%, *p* = 0.149), acute kidney failure (28.6% vs 21.4%, *p* = 0.230), stroke (3.9% vs. 1.9%, *p* = 0.380), reintervention rate (1.3% vs. 0%, *p* = 0.156) there was a trend towards a higher event rate in the bViV-TAVI group without reaching statistically significant differences.

Duration of the hospital stay (13.0 ± 7.2 vs 14.12 ± 9.8, *p* = 0.809) did not show statistically significant differences.

Regarding further peri-procedural complications, bViV-TAVI patients showed higher rates of a new permanent pacemaker implantation (20.8% vs. 9.1%), more bleeding events (46.75% vs. 19.5%, *p* < 0.001) and a trend towards a higher rate of vascular complications (23.4% vs. 10.4%, *p* = 0.09).

Post-procedural transvalvular mean pressure gradient was significantly higher (11.7 mmHg ± 5.6 vs. 10.0 mmHg ± 5.5, *p* = 0.02) in the bViV-TAVI group, whereas there was no difference concerning the frequency of PVL (Table 4).

**Table 4:** Outcome data

Features	Control	Absolut control	TAVI-VIV	Absolut ViV	*p*-value
New pacemaker	9.1%	14	20.8%	16	0.013
Acute kidney failure	21.4%	33	28.6%	22	0.230
Stroke	1.9%	3	3.9%	3	0.380
In-hospital death	2.5%	4	6.5%	5	0.149
VARC bleeding	19.5%	30	46.75%	36	< 0.001
VARC vascular complications	10.4%	16	23.4%	18	0.090
Discharge creatinine	1.0 ± 0.4		1.4 ± 1.2		0.003
Discharge Hb	11.0 ± 1.3		10.7 ± 1.3		0.089
Delta Pmean discharge (mmHg)	10.0 ± 5.5		11.7 ± 5.6		0.026
More than mild PVL	7.7%	12	7.8%	6	1.000
30-day-reintervention rate	0%	0	1.3%	1	0.156
30-day-endocarditis	0%	0	0%	0	
Duration hospital stay	14.12 ± 9.8		13.0 ± 7.2		0.809

Antithrombotic management was similar in both groups. The majority of patients received dual antiplatelet therapy in both groups (38.3% vs. 33.8%), followed by antiplatelet monotherapy (18.2% vs. 24.7%) if no anticoagulation was required. Otherwise, anticoagulation monotherapy or a combination of anticoagulation and platelet inhibitors was used.

After an average follow-up period of 4.9 ± 3.0 years all-cause mortality was significantly higher in the bViV -TAVI group (54.5% vs. 39.0%, *p* = 0.025).

In our study, in an average of 1.9% of the cases, the implantation of a second valve was performed. On a detailed examination we found that over the first 4 years (2011–2014) bViV-rates ranged from 3.5 to 4.8% with an average of 4.1%. For the next 4 years (2015–2018) the average bViV-rate sank to 1.8% with a further decrease during the last years (2019–2021) to an average of 1.3% (Supplementary material, Table S5).

### Predictors of all-cause mortality

The hazard ratio (HR) revealed that risk factors for all-cause mortality taking into consideration all patients (bViV-group and TAVI-group) are pre-existing atrial fibrillation or flutter [HR 2.300 (95% CI 1.468–3.602); *p* < 0.001], acute kidney failure [HR 2.069 (95% CI 1.349–3.172); *p* < 0.001] and the bViV-procedure [HR 1.679 (95% CI 1.086–2.596); *p* = 0.020] with the most impact of atrial fibrillation or atrial flutter (Supplementary material, Table S2).

By only taking into account the bViV-patients, conversion to surgery [HR 12.728 (95% CI 1.365–118.669); *p* = 0.044] and acute kidney failure [HR 2.096 (95% CI 1.021–4.303); *p* = 0.044] were independent predictors for all-cause mortality (Supplementary material, Table S3).

### Predictors of bViV-TAVI

Peripheral arterial disease [HR 4.128 (95% CI 1.648–10.343); *p* = 0.002] and a higher baseline mean pressure gradient [HR 1.027 (95% CI 1.008–1.047); *p* = 0.005] were identified as predictors for the implantation of a second valve (Supplementary material, Table S4).


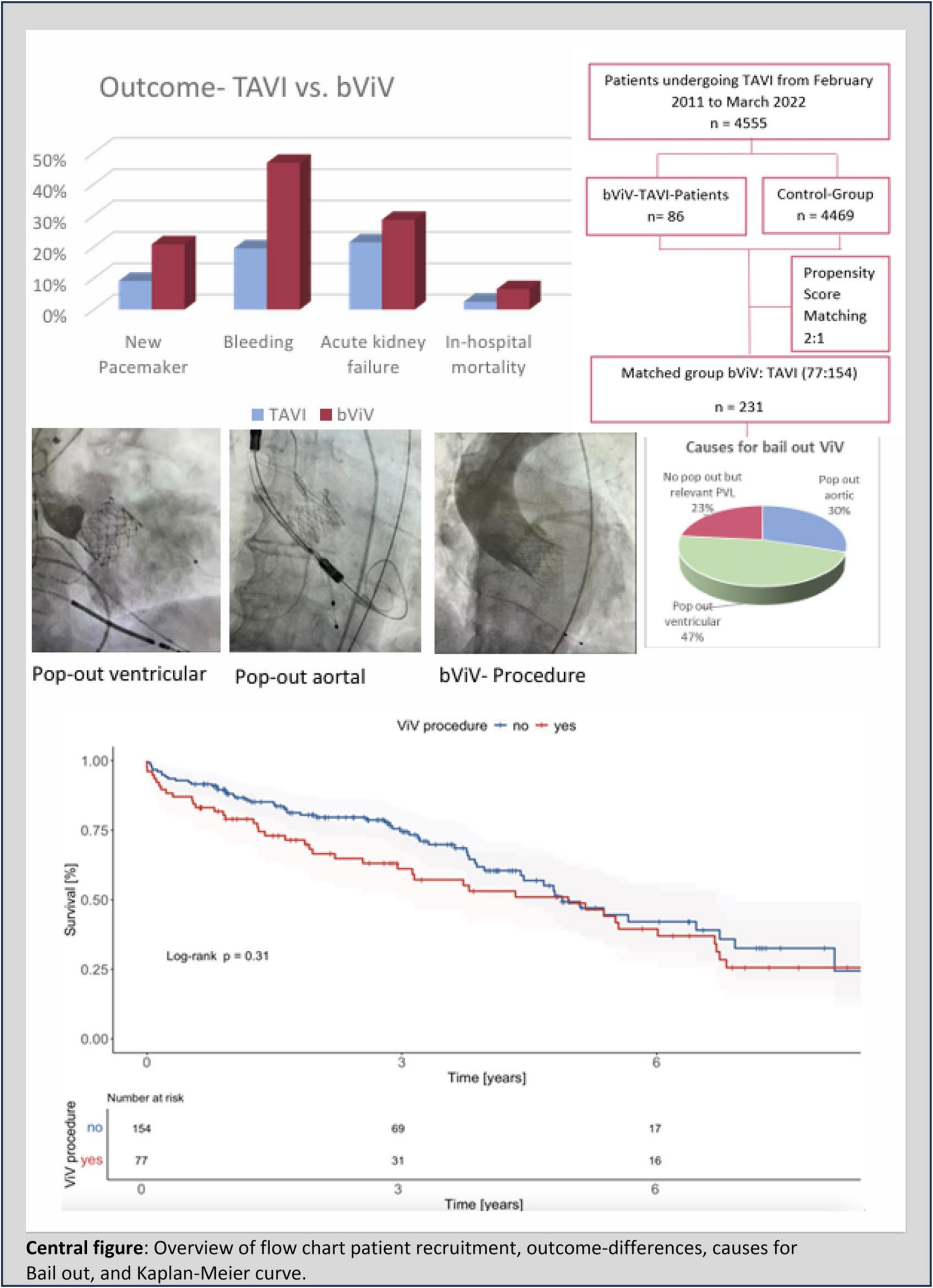


## Discussion

We objected to evaluating long-term mortality and periprocedural outcomes in patients who needed an acute unplanned second TAVI in the same procedure as a bail-out strategy by failed implantation of the first TAVI valve.

The main findings are:

1. Bail-out ViV-TAVI implantation is a rare event (1.9%).
2. TAVI embolization/migration is the main cause of bViV-TAVI followed by paravalvular leakage.
3. Patients with bail-out ViV-TAVI have a higher rate of periprocedural events and significantly worse long-term survival.

Antecedent reported incidences of TVEM ranging between 0.92 and 3.6%. Our study is with a bail-out ViV procedure-rate of 1.9% within this reported range [14, 18] with the highest rate for Evolute prosthesis, follow by Sapien and third Acurate. Predictors for bail-out ViV were described as an increased age and a smaller body mass index [14] whereas peripheral artery disease and higher baseline transvalvular gradient were identified as predictors in our cohort.

Pop-out occurred more frequently ventricular than aortal which could be attributed to the higher rate of self-expanding prosthesis in the first place as different distributions were detected when considering only balloon-expandable devices [19].

Of interest, bail-out ViV rates showed a significant decrease over time (Supplementary material, Table S5). These findings could be attributed to the growing operator's experience and the increased number of TAVI implantations over the years which underlines the implication of a diminishing tendency for TVEM with a center´s increasing experience described by Makkar et al. [19] and Frumkin et al. [18].

### Bail-out ViV and conversation rates

The conversion rate of the bViV group in our cohort was comparably low (2.6%, 2/77 patients) whereas most of the other studies reported higher conversation rates [13, 14, 18, 20]. The differences in the conversation rates could be caused by the fact that the indications for open surgery differed between the studies, for example including mandatory and optional indications [13] and different levels of implanters’ experience.

High mortality numbers were reported for patients undergoing conversion to surgery [20–22].

This is coherent with our study exposing a 30-day mortality rate of both cases undergoing conversion to surgery with 100%. These findings might be overestimated caused by a small number of cases.

### Periprocedural outcome

In the bViV-TAVI group periprocedural complications occurred more often which is in line with previous studies [13, 19]. In particular, the rate of bleeding events and vascular complications might be possibly explained by longer procedural times with extended manipulation by insertion of a second valve [13].

In accordance with other studies more contrast medium was used in the bViV-group and longer intervention times were found [13, 14, 19]. Nevertheless, there was no significant difference concerning the event of acute kidney failure in our patients.

#### Pacemaker and stroke rates

In line with existing data, we found a significantly increased rate of new pacemaker implantations (20.8% vs. 9.1%, *p* = 0.013) in the bViV-cohort [14, 19] that might be contributed to increased manipulation close to the annulus by implanting two valves within a short period of time as well as implanting the second valve deeper as recommended increasing the risk of conduction disturbances.

The stroke rate reported in various studies has shown a wide range [19, 20].

However, although a trend towards a slightly increased stroke rate in the bViV-group could be detected, the distinctness was not significantly different (3.9% vs. 1.9%, *p* = 0.380).

#### MPG and PVL

Contrary to the other studies [13, 19] we found a significantly higher mean aortic valve gradient in the bViV-group on discharge transthoracic echocardiography (11.7 ± 5.6 vs. 10.0 ± 5.5). Possibly caused by the fact that the secondary implanted valve in our study was mostly a balloon-expandable device with an intra-annular design. This finding has been described before [20]. However, this difference is clinically not meaningful.

In our cohort, 7.8% in the bViV—group had a more than mild PVL which is about the same amount as the patients in the TAVI group and was not predictable for a higher all-cause mortality.

#### In-hospital mortality

In-hospital mortality tended to be higher in the bViV group underlined by earlier findings [14, 19, 20, 23].

Surgical conversation as bail-out was a clear predictor of higher mortality as reported previously [13].

#### Long-term outcome

There are several long-term outcome studies for TAVI patients receiving a single TAVI prosthesis, especially in comparison to surgical treatment [4, 9, 24–26], but for patients who underwent an unplanned, acute, bail-out ViV-procedure the longest so far reported follow-up period was 2.2 years [14].

In our cohort, the mean follow-up was 4.9 years showing an increased risk of death for the bViV-patients independent from the reason for bViV implantation (all-cause mortality 50% for PVL and 56% for TVEM).

Long-term mortality for the TAVI-control group was comparable to other datasets including patients with intermediate surgical risk and a standard TAVI-implantation of a self-expanding or balloon-expandable valve with similar follow-up periods (SURTAVI trial, [26]); PARTNER 2 A Trial [4].

#### Risk factors

Our findings may emphasize that higher attention should be paid to patients with preexisting atrial fibrillation or flutter and patients developing acute kidney failure postprocedural as it was found to be a strong independent predictor for all-cause mortality as well in the total cohort as for the bViV-patients.

Coherent to previous findings the implantation of a second valve in the same procedure as a bail-out strategy was found to be an independent predictor of all-cause mortality [19].

## Limitations

Due to the retrospective and single center design the study typical limitations apply. To control for confounding baseline variables a propensity score adjusted analysis was used, but bias due to unknown or unmeasured confounders cannot be excluded.

Due to a relatively small number of bViV patients (bail-out rate of only one to two percent) the differences in secondary endpoints did not reach statistical significance, so the observed effects could be underestimated.

## Conclusion

In the rare event of an initially failed TAVI implantation, the implantation of a second valve during the same procedure as bail-out is a feasible alternative treatment option. However, a trend towards higher periprocedural complication rates and a significant increased long-term mortality has to be taken into account.

## Supplementary Information

Below is the link to the electronic supplementary material.

Supplementary file1 (DOCX 25 KB)
