# Supplementary material for: Long-term outcome of bail-out ViV-TAVI at index procedure
Source: Clin Res Cardiol. 2025 Apr 29;114(11):1445–54. doi: 10.1007/s00392-025-02640-5 (PMC12540624; doi:10.1007/s00392-025-02640-5)
Supplement: Supplementary file 1 — Supplementary file1 (DOCX 25 KB) [file 392_2025_2640_MOESM1_ESM.docx]

**SUPPLEMENTARY DATA**

**Long-term outcome of bail-out ViV-TAVI at index procedure**

Supplementary Table 1: Baseline features

| **Features** | **Control** | **Control**  **absolute** | **ViV** | **ViV absolute** | **p-value** |
| --- | --- | --- | --- | --- | --- |
| Patients |  | 4594 |  | 86 |  |
| Age (years) | 81.54 ± 6.1 |  | 81.6 ± 5.9 |  | 0.730 |
| Female | 52.5 % | 2414 | 48.8 % | 42 | 0.495 |
| Body mass index [kg/m²] | 27.22 ± 5.1 |  | 26.7 ± 4.6 |  | 0.482 |
| Hypertension | 90,6 % | 4112 | 96.5 % | 83 | 0.035 |
| COPD | 14.5 % | 667 | 12.8 % | 11 | 0.652 |
| *New York Heart Association class* |  |  |  |  |  |
| I | 2.4 % | 112 | 1.2% | 1 | 0.445 |
| II | 27.3 % | 1252 | 29.1% | 21 | 0.558 |
| III | 63.3 % | 2910 | 65.1% | 51 | 0.441 |
| IV | 7.0 % | 320 | 4.7% | 4 | 0.402 |
| Atrial fibrillation | 38.9 % | 1789 | 30.2% | 26 | 0.101 |
| Coronary artery disease | 55.4 % | 2683 | 66.3% | 57 | 0.142 |
| Previous cardiac surgery | 17.7 % | 815 | 25.6% | 22 | 0.06 |
| Peripheral artery disease | 13.6 % | 624 | 18.6% | 16 | 0.179 |
| Preexisting cerebrovascular disease | 6.0 % | 274 | 3.5% | 3 | 0.357 |
| Pre-existing Pacemaker/ ICD | 13.4 % | 616 | 7.0 % | 6 | 0.082 |
| STS Score [%] | 6.0 ±4.8 |  | 6.3 ±5.8 |  | 0.360 |
| Euroscore [%] | 21.4 ±15.9 |  | 22.3 ± 14.2 |  | 0.255 |
| Euroscore2 [%] | 6.5 ± 7.2 |  | 7.1 ± 8.4 |  | 0.130 |
| Pre Echo EF [%] | 50.9 ± 10.1 |  | 51.7 ± 9.6 |  | 0.912 |
| Pre Creatinine [mmol/l] | 1.3 ± 0.8 |  | 1.4 ± 0.9 |  | 0.313 |
| Pre CRP | 1.0 ± 2.1 |  | 1.2 ± 2.7 |  | 0.221 |
| Pre Hb | 12.5 ± 1.7 |  | 12.6 ±1.8 |  | 0.521 |
| Pre WBC | 7.8 ± 3.8 |  | 8.4 ± 3.3 |  | 0.093 |

Supplementary Table 2: Cox regression model for predictors of all-cause mortality all patients

| All-cause mortality | Hazard-Ratio | Confidence interval | p-value | Hazard-Ratio | Confidence interval | p-value |
| --- | --- | --- | --- | --- | --- | --- |
|  | Univariat | | | Multivariat | | |
| Atrial fibrillation/ atrial flutter | 2.320 | 1.534- 3.508 | <0.001 | 2.300 | 1.468 -3.602 | <0.001 |
| ANV | 2.089 | 1.371-3.185 | <0.001 | 2.069 | 1.349-3.172 | <0.001 |
| VARC vascular complication | 1.730 | 1.020-2.935 | 0.042 | 1.078 | 0.605-1.919 | 0.799 |
| VARC bleeding | 1.999 | 1.338-2.985 | <0.001 | 1.210 | 0.784-1.867 | 0.389 |
| Valve in Valve | 1.627 | 1.089-2.432 | 0.018 | 1.679 | 1.086- 2.596 | 0.020 |

Supplementary Table 3: Cox regression model for predictors of all-cause mortality bViV patients

| All-cause mortality bViV | Hazard-Ratio | Confidence interval | p-value | Hazard-Ratio | Confidence interval | p-value |
| --- | --- | --- | --- | --- | --- | --- |
|  | Univariat | | | Multivariat | | |
| Atrial fibrillation /flatter | 2.193 | 1.128-4.265 | 0.021 | 1.642 | 0.669-4.032 | 0.279 |
| ANV | 2.109 | 1.061-4.193 | 0.033 | 2.096 | 1.021-4.303 | 0.044 |
| Log_Euroscore % | 1.024 | 1.005-1.043 | 0.011 | 1.011 | 0.986-1.035 | 0.395 |
| BMI | 0.923 | 0.853-0.999 | 0.047 | 0.925 | 0.842-1.016 | 0.105 |
| Conversion | 11.655 | 1.283-105.919 | 0.029 | 12.728 | 1.365-118.669 | 0.026 |

Supplementary Table 4: Cox regression model for predictors of bViV

| bViV | Hazard Ratio | Confidence interval | p-value | Hazard Ratio | Confidence interval | p-value |
| --- | --- | --- | --- | --- | --- | --- |
|  | Univariat | | | Multivariat | | |
| Peripheral arterial disease | 2.419 | 1.101-5.317 | 0.028 | 4.128 | 1.648 | 10.343 |
| Pre echo mean pressure gradient | 1.022 | 1.004- 1.040 | 0.017 | 1.027 | 1.008 | 1.047 |

Supplementary Table 5: bViV-Rate over the years

| Jahr | Control-group | bViV-group | Percentage |
| --- | --- | --- | --- |
| 2011 | 115 | 4 | 3.5 |
| 2012 | 167 | 8 | 4.8 |
| 2013 | 230 | 10 | 4.3 |
| 2014 | 271 | 10 | 3.7 |
| 2015 | 345 | 5 | 1.4 |
| 2016 | 366 | 11 | 3.0 |
| 2017 | 391 | 8 | 2.0 |
| 2018 | 457 | 3 | 0.7 |
| 2019 | 553 | 6 | 1.1 |
| 2020 | 653 | 9 | 1.4 |
| 2021 | 711 | 9 | 1.3 |
